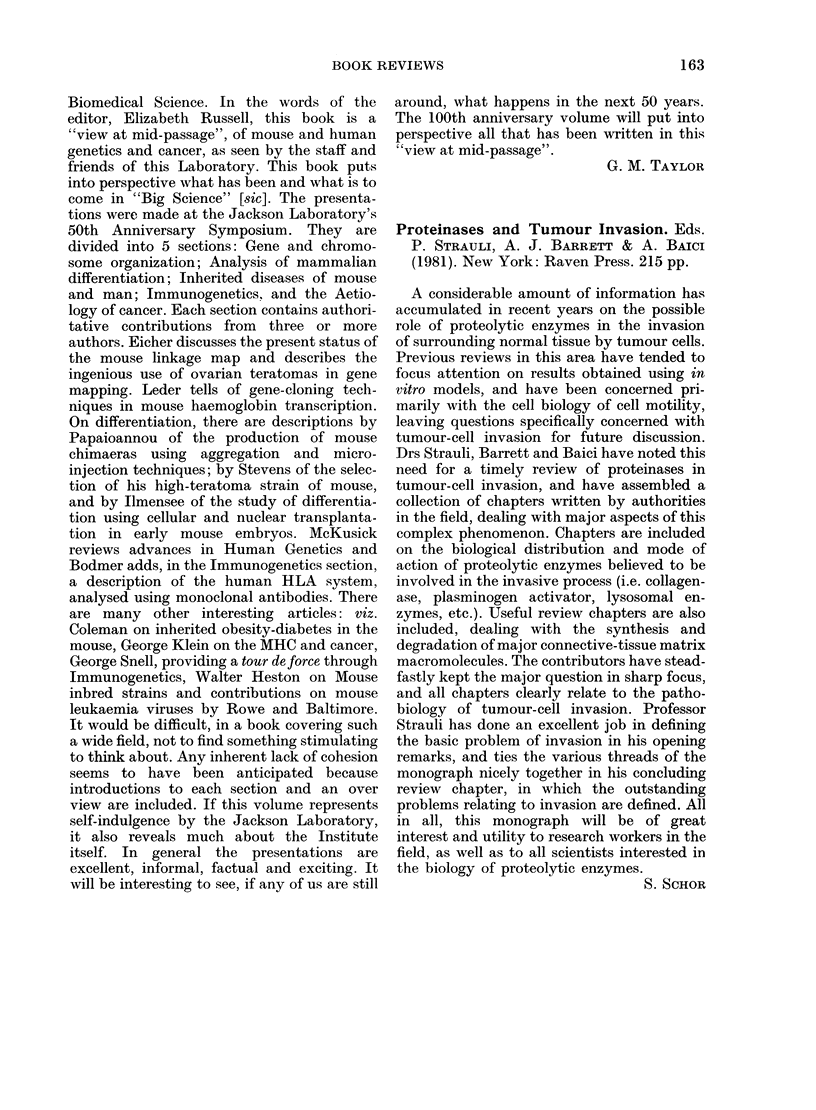# Proteinases and Tumour Invasion

**Published:** 1982-01

**Authors:** S. Schor


**Proteinases and Tumour Invasion.** Eds. P. Strauli, A. J. Barrett & A. Baici (1981). New York: Raven Press. 215 pp.

A considerable amount of information has accumulated in recent years on the possible role of proteolytic enzymes in the invasion of surrounding normal tissue by tumour cells. Previous reviews in this area have tended to focus attention on results obtained using *in vitro* models, and have been concerned primarily with the cell biology of cell motility, leaving questions specifically concerned with tumour-cell invasion for future discussion. Drs Strauli, Barrett and Baici have noted this need for a timely review of proteinases in tumour-cell invasion, and have assembled a collection of chapters written by authorities in the field, dealing with major aspects of this complex phenomenon. Chapters are included on the biological distribution and mode of action of proteolytic enzymes believed to be involved in the invasive process (i.e. collagenase, plasminogen activator, lysosomal enzymes, etc.). Useful review chapters are also included, dealing with the synthesis and degradation of major connective-tissue matrix macromolecules. The contributors have steadfastly kept the major question in sharp focus, and all chapters clearly relate to the pathobiology of tumour-cell invasion. Professor Strauli has done an excellent job in defining the basic problem of invasion in his opening remarks, and ties the various threads of the monograph nicely together in his concluding review chapter, in which the outstanding problems relating to invasion are defined. All in all, this monograph will be of great interest and utility to research workers in the field, as well as to all scientists interested in the biology of proteolytic enzymes.

S. Schor